# Systemic Treatment of Patients With Metastatic Breast Cancer: ASCO Resource–Stratified Guideline

**DOI:** 10.1200/GO.23.00285

**Published:** 2024-01-10

**Authors:** Sana Al Sukhun, Sarah Temin, Carlos H. Barrios, Nicoleta Zenovia Antone, Yanin Chavarri Guerra, Mariana Chavez-MacGregor, Rakesh Chopra, Michael A. Danso, Henry Leonidas Gomez, N’Da Marcelin Homian, Alaa Kandil, Benda Kithaka, Bogda Koczwara, Beverly Moy, Gertrude Nakigudde, Fernando Enrique Petracci, Hope S. Rugo, Nagi S. El Saghir, Banu K. Arun

**Affiliations:** ^1^ Al Hyatt Oncology Practice, Amman, Jordan; ^2^ American Society of Clinical Oncology, Alexandria, VA; ^3^ Oncoclinicas Group, Porto Alegre, Brazil; ^4^ Institutul Oncologic Prof Dr Ion Chiricuta, Cluj-Napoca, Romania; ^5^ Departamento de Hemato-Oncología, Instituto Nacional de Ciencias Médicas y Nutrición Salvador Zubirán, Mexico City, Mexico; ^6^ University of Texas MD Anderson Cancer Center, Houston, TX; ^7^ Gurugram, India; ^8^ Virginia Oncology Associates, Norfolk, VA; ^9^ Institute Nac de Enfermedades Neoplas, Surquillo, Peru; ^10^ CHU Treichville, Abidjan, Cote d’Ivoire; ^11^ Alexandria Comprehensive Cancer Center, Alexandria, Egypt; ^12^ Kilele Health Association, Nairobi, Kenya; ^13^ Flinders Medical Centre, Bedford Park, Australia; ^14^ Massachusetts General Hospital, Boston, MA; ^15^ Uganda Women's Cancer Support Organisation, Kampala, Uganda; ^16^ Instituto Alexander Fleming, Buenos Aires, Argentina; ^17^ University of California San Francisco, San Francisco, CA; ^18^ American University of Beirut, Beirut, Lebanon

## Abstract

**PURPOSE:**

To guide clinicians and policymakers in three global resource-constrained settings on treating patients with metastatic breast cancer (MBC) when Maximal setting–guideline recommended treatment is unavailable.

**METHODS:**

A multidisciplinary, multinational panel reviewed existing ASCO guidelines and conducted modified ADAPTE and formal consensus processes.

**RESULTS:**

Four published resource-agnostic guidelines were adapted for resource-constrained settings; informing two rounds of formal consensus; recommendations received ≥75% agreement.

**RECOMMENDATIONS:**

Clinicians should recommend treatment according to menopausal status, pathological and biomarker features when quality results are available. In first-line, for hormone receptor (HR)–positive MBC, when a non-steroidal aromatase inhibitor and CDK 4/6 inhibitor combination is unavailable, use hormonal therapy alone. For life-threatening disease, use single-agent chemotherapy or surgery for local control. For premenopausal patients, use ovarian suppression or ablation plus hormone therapy in Basic settings. For human epidermal growth factor receptor 2 (HER2)–positive MBC, if trastuzumab, pertuzumab, and chemotherapy are unavailable, use trastuzumab and chemotherapy; if unavailable, use chemotherapy. For HER2-positive, HR-positive MBC, use standard first-line therapy, or endocrine therapy if contraindications. For triple-negative MBC with unknown PD-L1 status, or if PD-L1–positive and immunotherapy unavailable, use single-agent chemotherapy. For germline *BRCA1*/*2* mutation–positive MBC, if poly(ADP-ribose) polymerase inhibitor is unavailable, use hormonal therapy (HR-positive MBC) and chemotherapy (HR-negative MBC). In second-line, for HR-positive MBC, Enhanced setting recommendations depend on prior treatment; for Limited, use tamoxifen or chemotherapy. For HER2-positive MBC, if trastuzumab deruxtecan is unavailable, use trastuzumab emtansine; if unavailable, capecitabine and lapatinib; if unavailable, trastuzumab and/or chemotherapy (hormonal therapy alone for HR-positive MBC).

Additional information is available at www.asco.org/resource-stratified-guidelines. It is ASCO's view that healthcare providers and system decision-makers should be guided by the recommendations for the highest stratum of resources available. The guideline is intended to complement but not replace local guidelines.

## INTRODUCTION

The purpose of this guideline is to provide expert guidance on the systemic treatment of metastatic breast cancer (MBC) to clinicians, public health leaders, patients, and policymakers in resource-constrained settings. This guideline's target population is adult patients with MBC in resource-constrained settings, and it focuses on medical treatment. This guideline is not intended for patients in Maximal settings, as described in Table 1.

**THE BOTTOM LINE**

**Systemic Treatment of Patients with Metastatic Breast Cancer: ASCO Resource–Stratified Guideline**

**GUIDELINE QUESTION**

What is the optimal treatment for patients diagnosed with metastatic breast cancer in resource-constrained settings?

**TARGET POPULATION**

Adult patients with metastatic breast cancer in resource-constrained settings.

**TARGET AUDIENCE**

Clinicians, public health leaders, patients, and policymakers in resource-constrained settings

**METHODS**

A multinational, multidisciplinary Expert Panel was convened to develop clinical practice guideline recommendations on the basis of an expert consensus process.

Author's note. It is the view of ASCO that health care providers and health care system decision-makers should be guided by the recommendations for the highest stratum of resources available. The guidelines are intended to complement but not replace local guidelines.

**RECOMMENDATIONS**

**General Notes**

1. Palliative care needs should be addressed for all patients at presentation of metastatic breast cancer (MBC), including situations in which no antineoplastic interventions are accessible.
2. Patients who are premenopausal can only receive aromatase inhibitors if accompanied by ovarian ablation or ovarian suppression.
3. Clinicians should recommend treatment according to pathological and biomarker features when quality (following established guidelines) testing results are available.
4. Cases should be discussed using a multidisciplinary approach with the core team including the surgeon, pathologist, oncologist, and radiation oncologist.

**First-Line**

**Hormone Receptor–Positive**

Assessment of menopausal status is critical; ovarian suppression or ablation should be provided to patients who are premenopausal. Patients whose tumors express any level of hormone receptors (HRs) should be offered hormone therapy. In Basic settings, if no immunohistochemistry testing is available, clinicians may presume HR positivity and offer tamoxifen in most cases.

For patients with HR-positive, human epidermal growth factor receptor 2 (HER2)–negative MBC, when non-steroidal aromatase inhibitors (AIs) and CDK 4/6 inhibitors are not available, use hormonal therapy alone. For life-threatening disease, clinicians may use single-agent chemotherapy; surgery may be used in cases in need of “salvage mastectomies” and for local control.

For patients with HR-positive, HER2-negative MBC who are premenopausal, ovarian suppression or ablation plus hormone therapy should be offered.

Patients with HR-positive, HER2-negative MBC for whom chemotherapy is offered, should be prescribed single-agent chemotherapy rather than combination chemotherapy, although combination regimens may be offered for highly symptomatic or life-threatening disease.

Patients with HR-positive MBC with disease progression on an endocrine agent who are postmenopausal may be offered treatment with either:

- endocrine therapy with or without targeted therapy or
- single-agent chemotherapy.

Patients with HR-positive MBC who are premenopausal without prior hormone therapy may be offered treatment with:

- Tamoxifen, or ovarian ablation or ovarian suppression alone, or sequential hormonal therapy, or non-steroidal AIs with ovarian ablation or ovarian suppression and CDK4/6 inhibitors in Enhanced settings.
- Tamoxifen, or ovarian ablation or ovarian suppression with hormonal therapy in Limited settings.
- Tamoxifen in Basic settings.

Patients with HR-positive MBC with disease progression on an endocrine agent who are premenopausal may be offered treatment with:

- Ovarian ablation or ovarian suppression with hormonal therapy or sequential hormone therapy in Enhanced settings.
- Alternative hormone therapy or surgery in Limited settings.
- Tamoxifen and bilateral oophorectomy in Basic settings.

**HER2-Positive**

HER2-targeted therapy is recommended for patients with HER2-positive advanced BC, except for those with clinical congestive heart failure or significantly compromised left ventricular ejection fraction, who should be evaluated on a case-by-case basis.

Trastuzumab, pertuzumab, and taxanes for first-line treatment are recommended. If pertuzumab isn't available, then clinicians may offer chemotherapy and trastuzumab in Enhanced settings. Chemotherapy may be offered in Limited settings.

For patients with HER2-positive and HR-positive MBC, various HER2-targeted therapies and chemotherapy or endocrine therapy, or chemotherapy alone or endocrine therapy alone may be offered depending on availability of anti-HER2 therapies. See Table 5 for special circumstances for this population.

**Triple-Negative**

Patients with triple-negative MBC that is known PD-L1–positive may be offered the addition of an immune checkpoint inhibitor to chemotherapy as first-line therapy in Enhanced settings; most patients with triple-negative MBC in Limited settings may be offered chemotherapy.

Patients with triple-negative, PD-L1–negative MBC should be offered single-agent chemotherapy rather than combination chemotherapy as first-line treatment.

For *BRCA1* or *BRCA2* mutation carriers with metastatic HR-negative, HER2-negative BC, poly(ADP-ribose) polymerase inhibitor (PARPi) therapy may be offered in Enhanced settings.

Patients with HR-positive MBC and known *BRCA* mutations and if PARPi therapy is not available, treatment options include hormonal therapy with or without ovarian ablation.

**Second-Line**

**HR-Positive**

In Enhanced settings, recommendations depend on prior treatment, for example, with prior endocrine therapy, clinicians may offer second-line endocrine therapy with or without targeted therapy (eg, CDK4/6 inhibitor or everolimus). In Limited settings with prior endocrine therapy, clinicians may offer second-line endocrine therapy if available, otherwise, they may offer chemotherapy.

**HER2-Positive**

HER2-targeted therapy should be given based on prior therapy and HR status. Trastuzumab deruxtecan or alternate HER2-targeted therapy regimens may be offered as second-line treatment depending on availability. In Limited settings, chemotherapy may be offered (with trastuzumab, if available). In Basic settings, if a patient has received prior treatment and medical treatment and pathology aren't available and has symptoms, clinicians may offer primary surgery for palliative reasons, including local control. If a patient finished trastuzumab-based adjuvant treatment less than 1 year before recurrence, offer second-line options. If more than 1 year before recurrence, offer first-line options.

**HR-Positive, *BRCA1*/*2* Mutations**

Patients with HR-positive MBC with germline *BRCA1*/*2* mutations no longer benefiting from endocrine therapy may be offered a PARPi rather than chemotherapy; chemotherapy may be offered if a PARPi is not available.

**Triple-Negative**

In second-line, with or without prior PD-L1 checkpoint inhibitors, clinicians may offer chemotherapy, if sacituzumab govitecan is unavailable.

Patients with triple-negative MBC with germline *BRCA1*/*2* mutations previously treated with chemotherapy may be offered a PARPi rather than chemotherapy.

**Third-Line**

**HER2-Positive**

In the third-line setting, clinicians should offer other HER2-targeted therapy combinations. (For patients with HER2-positive, HR-positive MBC, offer hormonal therapy with or without trastuzumab.)

**Triple-Negative**

In the third-line setting, triple-negative MBC may be eligible for PARPi (if germline *BRCA1*/*2* mutation status is known), if not available, then clinicians may offer chemotherapy and/or palliative care.

See Table 8 for other third-line regimens.

**ADDITIONAL RESOURCES**

More information, including a Data Supplement, slide sets, and clinical tools and resources, is available at www.asco.org/resource-stratified-guidelines. The Methodology Manual (available at www.asco.org/guideline-methodology) provides additional information about the methods used to develop this guideline. Patient information is available at www.cancer.net.

**ASCO believes that cancer clinical trials are vital to inform medical decisions and improve cancer care and that all patients should have the opportunity to participate.**

**TABLE 1:**
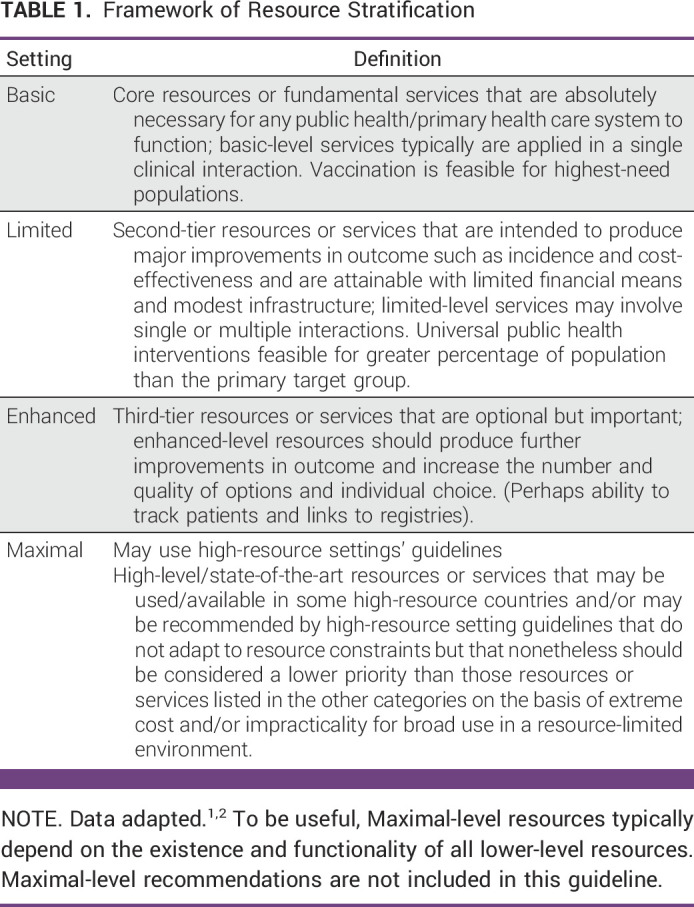
Framework of Resource Stratification

In 2020, BC was the most common malignancy worldwide (first most incident in 2020 per Cancer Today—IARC), surpassing lung cancer for the first time in years.^3,4^ It was also the fifth most frequent cause of cancer-related mortality and remains the leading cause of cancer mortality among women worldwide.^3,5^ Approximately 1.4 million of the 2.26 million new cases of breast cancer (BC)^3^ diagnosed in 2020 were made in low- and middle-income countries (LMICs; 62%), increasing the 5-year prevalence to 4.2 million (54%) of the worldwide 7.8 million cases. The proportion of deaths resulting from BC (72%) in LMICs was higher than their share of incidence (62%), indicating a higher case fatality rate (Tables 2 and 3).^8^ These statistics illustrate the importance of focusing on BC control as a major contributor to cancer morbidity and mortality in LMICs. Younger age at presentation is a unique aspect of BC diagnosis in LMICs.^8,9^ Two thirds of BC cases in Low– and Middle–Human Development Index (HDI) countries are diagnosed in patients younger than 50 years, as compared with one third of BC cases in High-HDI countries, with a median age at presentation being a decade younger than in High-HDI countries.^5,9^ BC in young women has been associated with unique biology, genetics, and inferior outcomes as compared with BC in older women.^10^ This unique aspect could partially contribute to the challenge of more advanced presentation as compared with high-income countries.^11^ Young age and dense breasts, even in the digital era, remain risk factors for false-negative mammography in patients with symptoms^12^; in addition, advanced stage may be driven by unique biology.^11^ Awareness campaigns are important to support early detection, but they can also play an important role in encouraging the population to proceed with diagnosis and treatment. As stated by Al-Sukhun et al in the ASCO Education Book 2022, “most of the time, patients associate expensive medication with dramatic benefit, even if the scientific evidence does not support these expectations. If they cannot afford those expensive medications, they decline treatment or shy away from seeking health care.”^4(p420)^ Awareness campaigns must address such expectations and educate patients on the benefit they may experience from standard surgery, radiation therapy, and affordable medications. Lack of access to high-end technology should not be associated with lack of benefit from treatment of patients with cancer.^8^ In resource-constrained settings, efficient allocation of resources mandates prioritization. Many cancer medicines included in the WHO Essential Medicines List (EML) are not available or are expensive in LMICs (see Appendix Table A4).^13,14^ The concept of resource limitations is no longer solely applied to countries defined by income according to the World Bank definition; it is a dynamic concept contingent on the stability of the society or the region of concern and the concept of human development indices.^15-18^ The provision of evidence-based and context-appropriate guidance for clinicians has been demonstrated to improve the outcome of patients with BC, supporting a public health approach to best inform clinical decisions in that specific setting.^19^

**TABLE 2:**
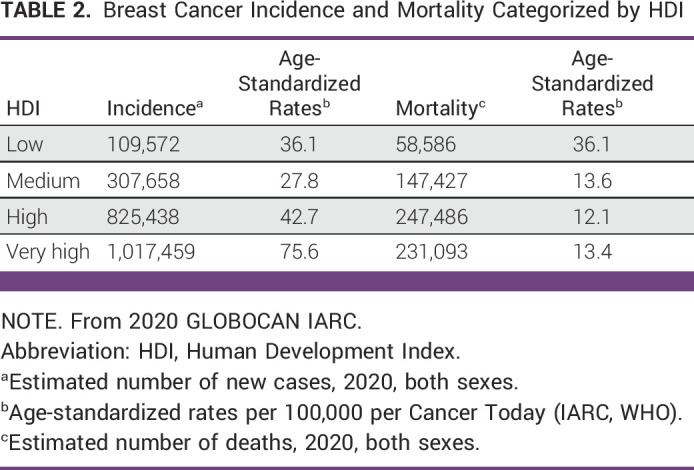
Breast Cancer Incidence and Mortality Categorized by HDI

**TABLE 3:**
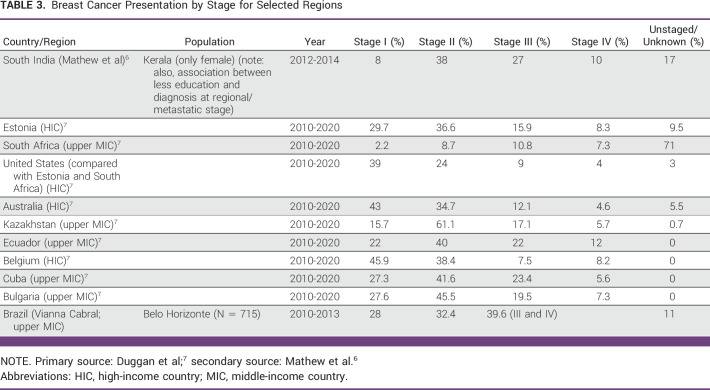
Breast Cancer Presentation by Stage for Selected Regions

Anatomic diagnosis is central to the diagnosis and treatment of malignancies at the individual patient level. Histopathologic evaluation assigns subtype and grade and identifies secondary prognostic features and molecular signals that predict prognosis and the choice of therapy for cancer. Pathology and laboratory medicine services face substantial implementation challenges in resource-constrained settings.^20^ Those include insufficient human capacity; inadequate infrastructure; inadequate consumables; inadequate education and training; and inadequate quality, standards, and accreditation.^20^ Therefore, recommendations on the basis of subtype identification should take into consideration the fact that accurate diagnostics might not be available in Basic settings and should help oncologists treat patients accordingly. The same limitations apply when considering surgical and radiation therapy services. However, there is extreme heterogeneity in access to these services in limited resource settings.^21,22^

Different regions of the world, both among and within countries, have variable access to diagnosis and treatment of BC. In addition, patients' access to medicines may change quickly. Patients with MBC ideally require the care of specialists including medical oncologists who have extensive training. However, outside of specialized centers within High-HDI regions, there is a paucity of specialty training with few clinicians available to skillfully manage these patients. Some of the presumptions inherent in this guideline include that chemotherapy is not available in Basic settings (Table 4). As a result of these disparities, the ASCO Resource–Stratified Guideline Advisory Group chose MBC as a priority topic for guideline development.

**TABLE 4:**
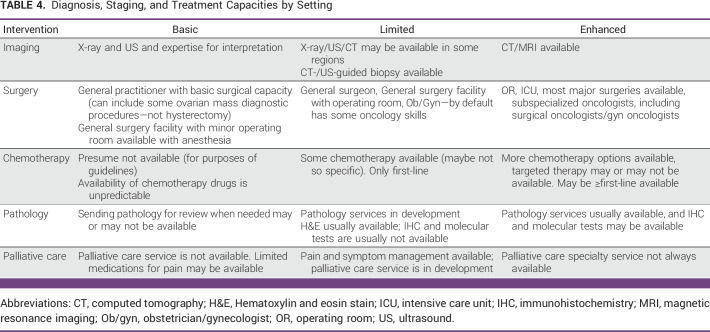
Diagnosis, Staging, and Treatment Capacities by Setting

ASCO has established a process for the development of resource-stratified guidelines,^23^ which includes mixed methods of evidence-based guideline development, adaptation of the clinical practice guidelines of other organizations, and formal expert consensus. This article summarizes the results of that process and presents resource-stratified recommendations (see the Results section).

Although this guideline refers to the sex of people at risk of BC as women, all people with breasts are at risk for BC and the guidelines apply uniformly to all such people. Therefore, despite limited data when it comes to BC in men, the Expert Panel recommends they have access to the same therapeutic options as patients born female as recommended in evidence-based guidelines. In instances in which the guideline draws on data on the basis of gendered research (eg, studies regarding women with BC), the guideline authors describe the characteristics and results of the research as reported.

In developing resource-stratified guidelines, ASCO has adopted its framework from the four-tier resource setting approach (Basic, Limited, Enhanced, Maximal; Table 1) developed by Breast Health Global Initiative and modifications to that framework on the basis of Disease Control Priorities 3.^1,2^ The framework emphasizes that variations occur not only between but also within countries with disparities, for example, between rural and urban areas.

*Full list of recommendations is available in corresponding Tables 5-7.^24^

**TABLE 5:**
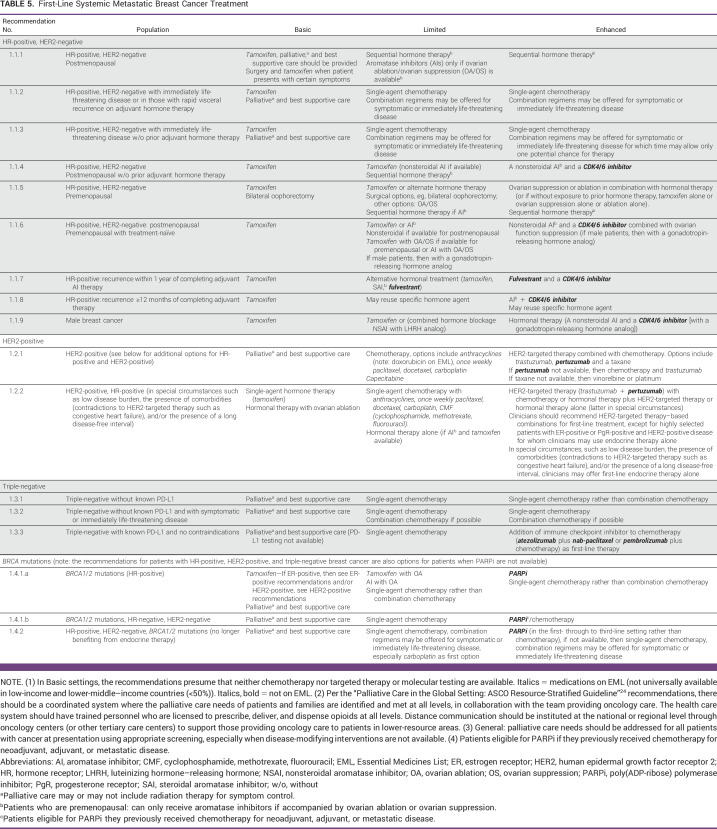
First-Line Systemic Metastatic Breast Cancer Treatment

**TABLE 6:**
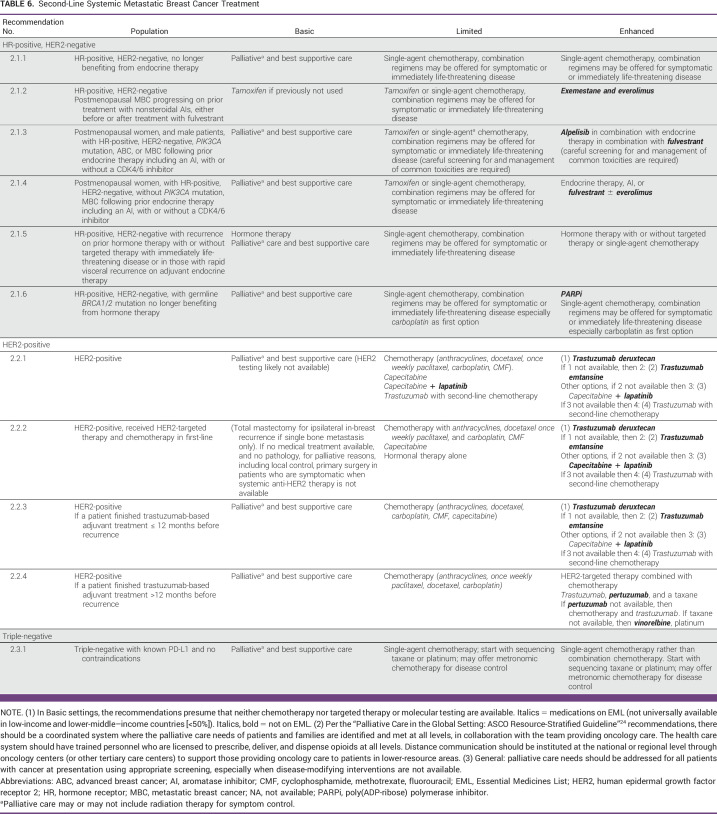
Second-Line Systemic Metastatic Breast Cancer Treatment

**TABLE 7:**
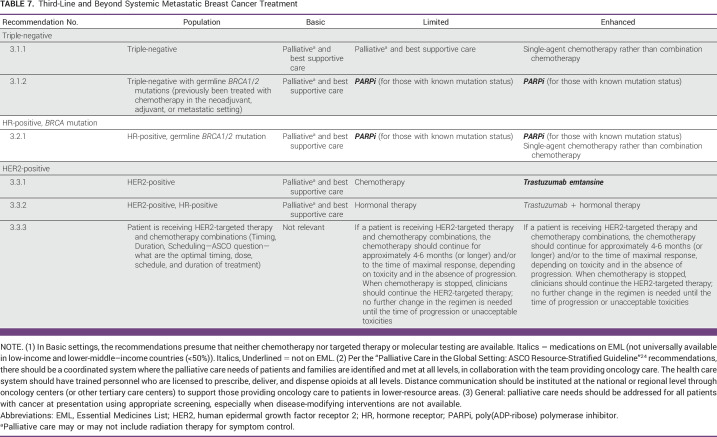
Third-Line and Beyond Systemic Metastatic Breast Cancer Treatment

**TABLE 8:**
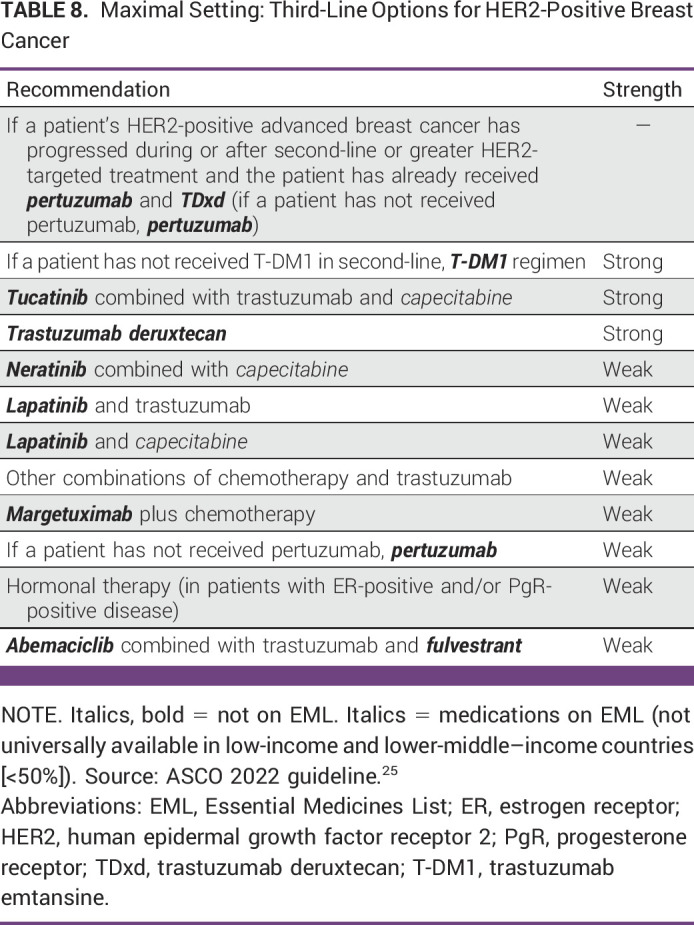
Maximal Setting: Third-Line Options for HER2-Positive Breast Cancer

## GUIDELINE QUESTIONS

This clinical practice guideline addresses the following overarching clinical question: What is the optimal treatment for patients diagnosed with metastatic breast cancer in resource-constrained settings (in three settings: Basic resource settings, Limited resource settings, and Enhanced resource settings)?

## METHODS

### Guideline Development Process

This formal consensus-based guideline product was developed by an international, multidisciplinary Expert Panel, which included two patient representatives and an ASCO guidelines staff member with health research methodology expertise (Appendix Table A1). The Expert Panel met via teleconference and corresponded through e-mail. On the basis of the consideration of the evidence, the authors were asked to contribute to the development of the guideline, provide critical review, and finalize the guideline recommendations. Members of the Expert Panel were responsible for reviewing and approving the penultimate version of the guideline, which was then circulated for external review and submitted to a peer-reviewed journal for editorial review and consideration for publication.

This guideline adaptation was also informed by the ADAPTE methodology and consensus methodology together as an alternative to de novo guideline development for this guideline. Adaptation of guidelines is considered by ASCO in selected circumstances when one or more quality guidelines from other organizations already exist on the same topic. The objective of the ADAPTE process is to take advantage of existing guidelines to enhance efficient production, reduce duplication, and promote the local uptake of quality guideline recommendations.^26^

ASCO's adaptation process begins with a literature search by ASCO guidelines staff to identify candidate guidelines for adaptation. Adapted guideline manuscripts are reviewed and approved by the ASCO Evidence-Based Medicine Committee (EBMC). The review includes content review completed by an Expert Panel (Appendix Table A1). All funding for the administration of the project was provided by ASCO. Further details of the methods used for the development of this guideline are reported in the ASCO Guidelines Methodology Manual (available at www.asco.org/guideline-methodology).

This guideline was partially informed by ASCO's modified Delphi Formal Expert Consensus methodology, during which the Expert Panel was supplemented by additional experts recruited to rate their agreement with the drafted recommendations. The entire membership of experts is referred to as the Consensus Panel (a list of members is available in Appendix Table A2). In round 1, a total of n = 30 respondents (11 of whom were on the Expert Panel) participated; in round 2, a total of n = 23 respondents (11 of whom were on the Expert Panel) participated. The guideline recommendations were crafted, in part, using the Guidelines Into Decision Support (GLIDES) methodology.^27^ The guideline recommendations were sent for an open comment period of 2 weeks, allowing the public to review and comment on the recommendations after submitting a confidentiality agreement. These comments were taken into consideration while finalizing the guideline. All ASCO guidelines are ultimately reviewed and approved by the Expert Panel and the ASCO EBMC before publication.

### Guideline Disclaimer

The clinical practice guidelines and other guidance published herein are provided by the ASCO, Inc to assist providers in clinical decision making. The information therein should not be relied upon as being complete or accurate, nor should it be considered as inclusive of all proper treatments or methods of care or as a statement of the standard of care. With the rapid development of scientific knowledge, new evidence may emerge between the time information is developed and when it is published or read. The information is not continually updated and may not reflect the most recent evidence. The information addresses only the topics specifically identified therein and is not applicable to other interventions, diseases, or stages of diseases. This information does not mandate any particular course of medical care. Further, the information is not intended to substitute for the independent professional judgment of the treating provider, as the information does not account for individual variation among patients. Recommendations specify the level of confidence that the recommendation reflects the net effect of a given course of action. The use of words like “must,” “must not,” “should,” and “should not” indicate that a course of action is recommended or not recommended for either most or many patients, but there is latitude for the treating physician to select other courses of action in individual cases. In all cases, the selected course of action should be considered by the treating provider in the context of treating the individual patient. Use of the information is voluntary. ASCO does not endorse third party drugs, devices, services, or therapies used to diagnose, treat, monitor, manage, or alleviate health conditions. Any use of a brand or trade name is for identification purposes only. ASCO provides this information on an “as is” basis, and makes no warranty, express or implied, regarding the information. ASCO specifically disclaims any warranties of merchantability or fitness for a particular use or purpose. ASCO assumes no responsibility for any injury or damage to persons or property arising out of or related to any use of this information or for any errors or omissions.

### Guideline and Conflicts of Interest

The Expert Panel was assembled in accordance with ASCO's Conflict of Interest Policy Implementation for Clinical Practice Guidelines (“Policy,” found at http://www.asco.org/guideline-methodology). All members of the Expert Panel completed ASCO's disclosure form, which requires disclosure of financial and other interests, including relationships with commercial entities that are reasonably likely to experience direct regulatory or commercial impact as a result of promulgation of the guideline. Categories for disclosure include employment; leadership; stock or other ownership; honoraria, consulting or advisory role; speaker's bureau; research funding; patents, royalties, other intellectual property; expert testimony; travel, accommodations, expenses; and other relationships. In accordance with the Policy, the majority of the members of the Expert Panel did not disclose any relationships constituting a conflict under the Policy.

## RESULTS

### Literature Search

The recommendations were developed through a review of existing Maximal setting ASCO-published guidelines^25,28-33^ and clinical experience of the panel of experts. The methods of development of each systematic review–based ASCO guideline are available in each publication. A total of four ASCO guidelines on medical treatment of patients with metastatic breast cancer (MBC) were found. The Expert Panel was aware of three rapid updates published in 2022;^31,32,34^ however, since this resource-stratified guideline development started before those publications, the recommendations in this guideline do not necessarily reflect those updates (which are relevant only to Maximal settings).

These ASCO Maximal setting guidelines cover treatment of patients with MBC, both female and male, and with the following subtypes: (1) hormone receptor (HR)–positive, human epidermal growth factor receptor 2 (HER2)–negative, (2) HER-negative and either endocrine-pretreated or HR-negative (the latter referred to triple-negative in this guideline), and (3) HER2-positive.

The ASCO Expert Panel reviewed these guidelines; Appendix Table A3 lists links to the guidelines. The Expert Panel used these guidelines, literature suggested by the Expert Panel, and clinical experience as guides. The Expert Panel acknowledges the effort put forth by the authors and ASCO to produce evidence-based guidelines informing practitioners and institutions who provide care to patients with MBC.

In addition, the Panel was surveyed on the availability of ASCO-recommended interventions in their settings. While the sample was small, the results showed that the majority had access to hormonal therapy (including aromatase inhibitors [AIs]) and ovarian suppression or ablation; the following are not available in some settings: nonsteroidal AIs, fulvestrant, CDK4/6 inhibitors, pertuzumab, immunotherapy, poly(ADP-ribose) polymerase inhibitors (PARPis), *PIK3CA*-targeted therapy, sacituzumab govitecan, everolimus, trastuzumab deruxtecan, and trastuzumab emtansine (T-DM1). In addition, this guideline refers to a publication from Fundytus et al^35^ on “Access to cancer medicines deemed essential by oncologists in 82 countries: an international, cross-sectional survey;” while this survey was conducted in 2020, it does provide some additional context on clinicians' perceptions of availability.

Furthermore, the Panel sought feedback from international members and their colleagues on the availability of antineoplastic therapy for patients with MBC in resource-constrained settings, LMICs, and low- and middle-income regions during winter 2022-2023. Respondents generally believe that most cytotoxics, hormonal therapies, and earlier anti-HER2 (trastuzumab and occasionally pertuzumab) therapies are available for most individuals through public or private means. Respondents generally believe that eribulin, fulvestrant, nonsteroidal AIs, some CDK4/6 inhibitors, T-DM1, trastuzumab deruxtecan, tucatinib, alpelisib, sacituzumab govitecan, everolimus, and checkpoint inhibitors are not available for most individuals through public or private means.

## SUMMARY OF ADAPTED GUIDELINES

### Guidelines on Treatment of Patients Diagnosed With MBC

The Expert Panel identified clinical questions and/or categories within the adapted guidelines that would potentially match the ASCO clinical questions for resource-constrained settings. All the guidelines were developed on the basis of patients in Maximal settings, and therefore, the Expert Panel had to review and adapt the recommendations for resource-constrained settings on the basis of experience in resource-constrained settings and then validate the recommendations by formal consensus. The target populations were all in Maximal settings and included patients with MBC.

All the ASCO guidelines this guideline adapted used systematic review–based methods. The key evidence for the guidelines included systematic reviews, randomized clinical trials, clinical experience, and informal consensus. Therefore, many recommendations in this ASCO guideline were informed by this variety of expert-reviewed data and then validated by Formal Consensus.

The outcomes and end points in most studies reviewed by the adapted guidelines included efficacy (including overall survival [OS] and progression-free survival [PFS]), quality of life, and safety or adverse events.

### Results of ASCO Methodological Review

Because this guideline is based on ASCO guidelines, methodological review was not conducted.

## SELECTED RECOMMENDATIONS

The recommendations were developed by a multinational, multidisciplinary group of experts using evidence from the existing guidelines and clinical experience as a guide. The ASCO Expert Panel underscores that health care practitioners who implement the recommendations presented in this guideline should first identify the available resources in their local and referral facilities and endeavor to provide the highest level of care possible with those resources. ASCO Resource–Stratified Guidelines acknowledge that clinicians and medical institutions in resource-constrained settings are striving to provide more effective medications, human and material infrastructure, as well as interact with policymakers. The authors would like to make some general points applying to recommendations throughout this guideline: outcomes should be balanced with quality of life including financial toxicity; recommendations are made regarding what is feasible in resource-constrained settings in this publication.

Due to the large breadth of recommendations, the Panel elected to discuss selected areas.

### Overarching Clinical Questions on Patients With HR-Positive, HER2-Negative MBC

Recommendations 1.1.1-1.1.9 (first-line), 2.1.1-2.1.6 (second-line), 3.2.1 (patients with HR-positive, *BRCA1*/*2* mutation–positive MBC included in a separate section).

#### Discussion

The first set of treatment recommendations (Table 5) state that clinicians should recommend treatment with systemic therapy according to pathological and biomarker features when the results of biomarker testing and pathology are available. Basic immunohistochemistry (estrogen receptor [ER], progesterone receptor [PgR], and HER2) is an important step in characterizing the disease and should be sought when possible in every scenario, as this will critically inform treatment decisions.

#### HR-Positive

Using non-steroidal AIs and CDK4/6 inhibitors in the first-line setting is supported by strong evidence, with both PFS and OS benefits as compared with AIs alone according to recent ASCO guidelines. Prior ASCO guidelines also recommended AIs alone, which also provides patient benefit. For patients unable to access CDK4/6 inhibitors, the use of AI alone or tamoxifen is an alternative in Limited resource settings. In clinical presentations with high symptom burden and life-threatening disease (ie, visceral crises), single-agent chemotherapy, if available, and even surgery are acceptable treatment options. For patients who are premenopausal, ovarian suppression or ablation plus hormonal therapy, if available, should be discussed. Surgical ablation or radiotherapy may be recommended whenever feasible as alternatives to medical ovarian suppression.

##### Basic resource settings.

Patients should be offered tamoxifen and palliative care according to ASCO Palliative Care Guidelines and, when possible, be referred for treatment in less resource-constrained medical settings. The goal of palliative care is to prevent patient suffering. In patients progressing on tamoxifen, an AI, and later, where systemic chemotherapy is available, single-agent palliative chemotherapy can be offered.

##### Limited resource settings.

For patients with HR-positive MBC, offering tamoxifen or an AI (for patients who are postmenopausal) is appropriate even when there is limited access to combination therapy with CDK4/6 inhibitors to avoid interrupted supply. Sequencing AI after disease progression on tamoxifen is also appropriate. Single-agent palliative systemic chemotherapy should be offered when available in patients whose disease is progressing on endocrine therapy.

##### Maximal and Enhanced resource settings.

A combination of endocrine therapy with an AI and a CDK4/6 inhibitor should be the first-line treatment for patients with metastatic HR-positive BC (plus ovarian suppression or ablation for patients who are premenopausal). Further treatment lines should follow standard guidelines exploring hormonal agents while there is evidence of endocrine sensitivity. Once resistance to endocrine alternatives is established, chemotherapies and targeted treatments in selected patients (ie, patients whose cancers are *PI3KCA* or *BRCA1*/*2* mutated) are indicated.

#### Further Discussion

##### Basic resource settings.

Many of the recommendations for patients with HR-positive, HER2-negative MBC emphasize offering tamoxifen in the Basic setting. Since most patients with BC have cancers that are HR-positive,^29^ the guideline recommends tamoxifen as a reasonable alternative. While the critical importance of basic immunohistochemistry, the Panel acknowledges that in some Basic settings, HR testing may not be available. As the potential harms of tamoxifen are relatively low, the Panel determined it offering a treatment option for patients in this situation was important. Tamoxifen is the historical mainstay of treatment for patients with HR-positive BC and is a recommended option in ASCO's 2016 guideline and re-affirmed in 2021. However, even tamoxifen may not be available (according to the global survey by Fundytus et al,^35^ it was universally available to 36% of oncologists in LMICs, and deemed substantial out-of-pocket expenses by 38% of respondents.) Referring the patients to higher-resource settings, if possible, is recommended.^36^

For patients who are premenopausal, tamoxifen plus ovarian suppression (surgical oophorectomy or radiotherapy ablation, when available) should be recommended rather than medical ovarian suppression strategies. Only surgeons with gynecologic surgical expertise should perform oophorectomies.^37^

##### Limited resource settings.

In Limited resource setting recommendations for patients with HR-positive, HER2-negative MBC, sequencing tamoxifen with AIs, and ovarian ablation or suppression in patients who are premenopausal should be discussed. Chemotherapy for patients with resistance to hormonal treatments is appropriate when available. In recommendations 1.1.2, 1.1.3, 2.1.2, and 2.1.5, chemotherapy is an option in the Maximal setting guidelines. In recommendations 2.1.2, 2.1.3, and 2.1.4, the Maximal setting–recommended medical options are not likely available to most patients in the Limited settings, therefore, chemotherapy is an option.

##### Enhanced resource settings.

All the recommendations for HR-positive, HER2-negative, *BRCA1*/*2*-negative, or unknown correspond to the ASCO Maximal setting recommendations. In situations where patients were previously treated with hormonal adjuvant therapy, it is important to consider the timing of the recurrence or diagnosis of the metastatic disease. Patients with an early recurrence (in the first 2 years of adjuvant hormonal therapy) will likely have endocrine-resistant disease. Patients with a diagnosis of metastatic disease after 12 months of completion of their adjuvant therapy should be noted as more endocrine sensitive and more likely to respond to further hormonal alternatives.

### HER2-Positive

Recommendations 1.2.1-1.2.2, 2.2.1-2.2.4, 3.3.1-3.3.3.

#### Discussion

The treatment of patients with MBC is also contingent on the HER2 status of the patient's cancer. It is outside the scope of this resource-stratified guideline to discuss tissue testing. However, quality HER2 testing, such as that recommended by the College of American Pathologists (CAP)-ASCO,^38,39^ is unlikely to be available in more resource-constrained settings. In a survey of 191 providers, including 153 from areas termed LMICs in this publication, about 30% of providers could access HER2 testing in private, but not public settings.^40^ (While trastuzumab is currently on the EML; is not available in the public systems everywhere [note: according to Fundytus et al,^35^ it is only 15% universally available.])

In Basic and Limited resource scenarios, basic targeted anti-HER2 therapies such as trastuzumab are likely not available. In some settings, some of these agents may be available to patients with private health insurance in some resource-constrained regions. In cases where trastuzumab is not available, combination chemotherapy should be discussed. Sequencing single-agent chemotherapies should be the best alternative for patients whose disease is progressing. In Enhanced resource settings, single-agent chemotherapy in combination with available anti-HER2 therapy should be offered.

#### HER2-Positive First-Line

Recommendation 1.2.1. The guideline recommends a tiered approach if the ASCO-recommended agents are not available. In Enhanced settings, if pertuzumab isn't available, trastuzumab and chemotherapy or chemotherapy alone are acceptable, as these agents were used historically before the advent of targeted therapy. In addition, navelbine can be used with trastuzumab in the frontline setting if taxanes are not available. The efficacy of navelbine in this setting has been demonstrated in the HERNATA study with similar response rate and OS, but less adverse events.^41^ Before this study, vinorelbine plus trastuzumab was presumed to be less effective than docetaxel plus trastuzumab. There was no difference in efficacy and fewer adverse events in the vinorelbine arm compared to the control arm.

#### HER2-Positive Second-Line

Recommendations 2.2.1, 2.2.2, 2.23, and 2.2.4. For second-line therapy, especially where the ASCO maximal guideline recommends trastuzumab deruxtecan, a tiered approach is recommended for the Enhanced setting depending on what is available. For example, the most recent HER2-positive guideline (as of this writing) recommended trastuzumab deruxtecan; the previous Maximal setting guideline^42^ recommended T-DM1, consequently in Enhanced settings, if trastuzumab deruxtecan is not available, then T-DM1 is recommended, then if T-DM1 is not available, capecitabine and lapatinib are recommended, and if neither are available, then clinicians may offer trastuzumab and chemotherapy. This is the same for recommendation 2.2.2. Recommendation 2.2.4 also provides an additional chemotherapy option in Enhanced settings.

#### Surgery of the Primary Tumor

Surgery for a patient's primary tumor is an option recommended for patients with HER2-positive tumors who received HER2-targeted therapy and chemotherapy in the first line. The following section is not based on the ASCO Maximal setting guidelines, but rather literature suggested by the Panel. A separate systematic review was not conducted for this guideline.

The role and potential benefit of removing the primary tumor continue to remain unclear for all patients with advanced-stage BC.^43^ Previous studies have provided mixed results about the survival benefit from surgical excision. Prospective trials failed to resolve whether locoregional therapy is^44,45^ or is not^46,47^ associated with a survival advantage in MBC. Those prospective trials were performed prior to routine assessment of HER2 status. These studies have limitations, including lack of standard randomization by biomarker status, leading to more favorable tumor subtypes in the surgically treated group in the trial demonstrating survival benefit with surgery,^44^ while the trial led by Badwe et al^47^ did not utilize HER2-targeted therapy in patients with HER2-positive disease. A 2010-2012 retrospective cohort with data from 3,231 women in the National Cancer Database with HER2-positive MBC compared those who did and did not undergo definitive breast surgery showed surgery was associated with a 44% reduction in the risk of death.^48^ In addition, a recent systematic review and meta-analysis showed benefit in patients who had limited disease burden, only one metastatic site or bone-only metastasis, or with negative margin at surgery, especially among premenopausal patients.^45,49^ Those were consistent with the results reported by Soran et al, where a benefit was also described among patients with ER-positive disease.^44^ Therefore, primary tumor surgery in appropriately selected patients with de novo stage IV BC controls locoregional progression, but discussion continues regarding its impact on OS in patients with oligometastatic or bone-only disease. Until the Expert Panel has further prospective data, especially when medical therapy resources are constrained, surgery of the primary tumor in appropriately selected patients with limited disease burden, bone-only disease, and ER-positive and/or HER2-positive disease, who can attain negative margin on surgery especially those younger than 55 years, is recommended. The Panel acknowledges the controversy surrounding this recommendation, therefore, advise discussion with the patient, emphasizing the palliative benefit and the potentially positive impact considering the data. In addition, the Expert Panel suggests palliative mastectomy for patients with bleeding or progressively ulcerating tumors in spite of available therapy, especially in Basic or Limited settings; whenever radiation therapy is not available.

### Germline (g) *BRCA* Mutation–Positive (m+)

Recommendations 1.4.1 a, 1.4.1 b, 1.4.2, 2.1.6, 3.2.1.

#### Discussion

For recommendation 1.4.1 a, the ASCO Maximal setting guideline recommends PARPi, which are not recommended in Basic or Limited settings. *BRCA* testing with or without genetic counseling is likely not available^37^ in Basic and Limited resource settings and many patients will remain undiagnosed. PARPi are not offered unless there is a known g*BRCA* mutation and patients have received chemotherapy in neoadjuvant, adjuvant, or metastatic setting. In addition, treatment with PARPi is most likely not available in these settings. For patients with known g*BRCA* mutations where PARPi are not available, treatment options for patients with HR-positive disease include hormonal therapy with or without ovarian ablation. For patients with endocrine-refractory disease (single-agent or combination) chemotherapy (recommendations 1.4.2, 2.1.6) is an option.

### Triple-Negative

Recommendation 3.1.2. For patients with triple-negative MBC, that is g*BRCA*-mutated, who have received prior chemotherapy, third-line and beyond PARPi is recommended in Limited and Enhanced settings as this provides the best chance of increased PFS and response rates as described in the Moy et al guideline.^30^

### Further Discussion

Radiation and surgical treatment options and government-funded screening programs are dependent on fixed infrastructure and thus face tight resource constraints. Access to medicines, in particular simpler medicines that do not cause cytopenias, may be dependent on an individual's ability to pay and that may change. When it comes to medication prescribing, the resource setting can fluctuate over time, with geographic and individual circumstances, and the Panel acknowledges this dynamism, as well as clinicians' aspirations to reach best practice. Additional discussion of these issues is available in a companion article.

## SPECIAL COMMENTARY

### Pathology

Pathology is an important part of diagnosing the type of MBC and guiding management of patients with this disease. There are variable availability and financing for pathology services around the world. In some regions, clinicians might even have to make diagnoses without pathology. ASCO Resource–Stratified Guidelines use the capacity framework in Table 4 to guide pathology recommendations. As resource-constrained regions develop pathology services, the Expert Panel offers some suggestions specific to MBC in this Special Commentary section.

The clinical presentation and imaging findings of both benign tumors and other malignancies that can involve the breasts may be similar to or mimic those of MBC. Consequently, a histopathologic diagnosis should be undertaken before definitive treatment. Usually, routine histologic processing of formalin-fixed tissue is sufficient for pathologic diagnosis. Immunohistochemical studies are important in BC diagnosis and do provide additional confirmatory evidence and predictive and prognostic information. In some Limited and Enhanced settings, tissue can be sent to pathology for cell block in major cities. As they become more widely available, recent technological advances such as digital pathology may make pathologic diagnosis available to a larger proportion of patients. However, the Panel recognizes that these technologies are still far from reaching Basic resource settings and most Limited resource scenarios. Pathology laboratories are of variable quality, and investments in pathology have an impact not only in BC diagnosis but also in cancer management and should be seen as a priority in resource allocation and national cancer plans.

**ASCO believes that cancer clinical trials are vital to inform medical decisions and improve cancer care and that all patients should have the opportunity to participate. The expansion of oncology clinical trials in Limited and Enhanced settings is a global oncology priority.**

## LIMITATIONS OF THE RESEARCH AND FUTURE DIRECTIONS

There are limitations on the evidence to inform some of the recommendations, because of many recognizable factors, such as prioritization of patient care and limited funding and infrastructure for research in this subject.

When the optimal standard treatments are not available, where there are accessible established regional cancer centers, physicians may refer patients to them, where they might have access. The Expert Panel recognizes that for large parts of the world, there are not established national, let alone regional cancer centers.

Important limitations include insufficient research conducted in resource-constrained settings, lack of conclusive information on primary and preventive screening, and lack of published data on MBC management adapted to resource-constrained settings. Expert recommendations for resource-constrained settings should account for differential access to chemotherapy across Basic and Limited resource settings. A shortage in human resources of trained oncologists has led to task-shifting with variation in skill sets among general practitioners, general surgeons, and oncologists able to manage patients with MBC.

There is a significant need to further MBC research in resource-constrained settings, considering issues of surgery and chemotherapy access, treatment efficacy, and cost-effectiveness. The paucity of cancer research in limited resource settings needs further investigation, which can be achieved through collaborative research. The use of targeted therapy and immunotherapy for patients with MBC is actively under investigation, and further guidelines will include updates. Further limitations are listed in Table 9.

**TABLE 9:**
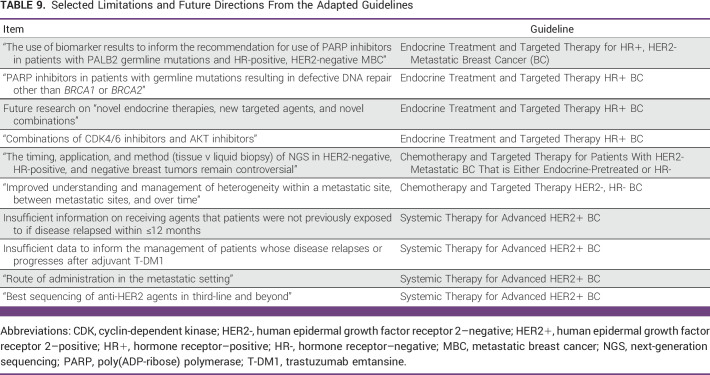
Selected Limitations and Future Directions From the Adapted Guidelines

## EXTERNAL REVIEW AND OPEN COMMENT

The draft recommendations were released to the public for open comment from April 12 through April 26, 2023. Response categories of “Agree as written,” “Agree with suggested modifications,” and “Disagree. See comments” were captured for every proposed recommendation with 13 written comments received. A total of 81%-100% of the 13 respondents either agreed or agreed with slight modifications to the recommendations, and 0%-19% of the respondents disagreed. Expert Panel members reviewed comments from all sources and determined whether to maintain original draft recommendations, revise with minor language changes, or consider major recommendation revisions. All changes were incorporated before EBMC review and approval.

The draft was submitted to two external reviewers with content expertise; two completed the reviews. It was rated as high quality, and it was agreed that it would be useful in practice. Review comments were reviewed by the Expert Panel and integrated into the final manuscript before approval by the EBMC.

## GUIDELINE IMPLEMENTATION

ASCO guidelines are developed for implementation across health settings. Barriers to implementation include the need to increase awareness of the guideline recommendations among frontline practitioners and patients with MBC and to provide adequate services in the face of limited resources. The guideline Bottom Line Box was designed to facilitate implementation of recommendations. This guideline will be distributed widely, including through many forms of ASCO communications and the ASCO website.

## ADDITIONAL RESOURCES

Additional information including a Data Supplement, evidence tables, and clinical tools and resources can be found at www.asco.org/resource-stratified-guidelines. Patient information is available there and at www.cancer.net.

## GENDER-INCLUSIVE LANGUAGE

ASCO is committed to promoting the health and well-being of individuals regardless of sexual orientation or gender identity.^50^ Transgender and nonbinary people, in particular, may face multiple barriers to oncology care including stigmatization, invisibility, and exclusiveness. One way that exclusiveness or lack of accessibility may be communicated is through gendered language that makes presumptive links between sex and anatomy.^51-54^ With the acknowledgment that ASCO guidelines may affect the language used in clinical and research settings, ASCO is committed to creating gender-inclusive guidelines. For this reason, guideline authors use gender-inclusive language whenever possible throughout the guidelines. In instances in which the guideline draws upon data on the basis of gendered research (eg, studies regarding women with BC), the guideline authors describe the characteristics and results of the research as reported.

**RELATED ASCO GUIDELINES**

**Resource-Stratified Guidelines**

• Palliative Care in the Global Setting^24^ (http://ascopubs.org/doi/10. 1200/JGO.18.00026)

**Non–Resource-Stratified Guidelines**

• Integration of Palliative Care into Standard Oncology Practice^55^ (http://ascopubs.org/doi/10.1200/JCO.2016.70.1474)
• ASCO Breast Cancer Guidelines (www.asco.org/breast-cancer-guidelines)
• Patient-Clinician Communication^56^ (http://ascopubs.org/doi/10.1200/JCO.2017.75.2311)

## Figures and Tables

**TABLE A1:**
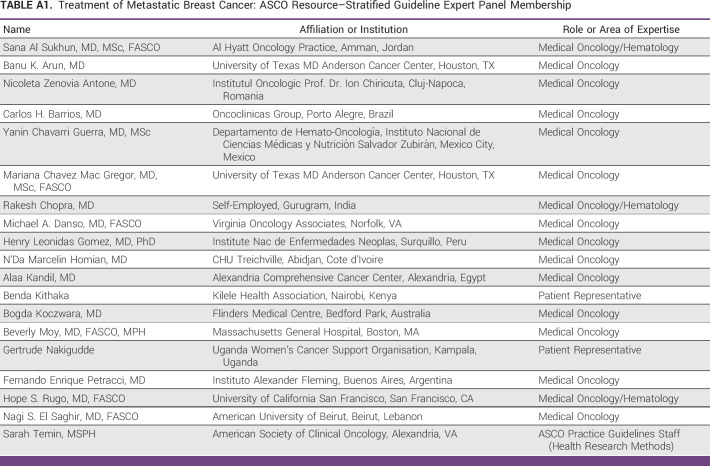
Treatment of Metastatic Breast Cancer: ASCO Resource–Stratified Guideline Expert Panel Membership

**TABLE A2:**
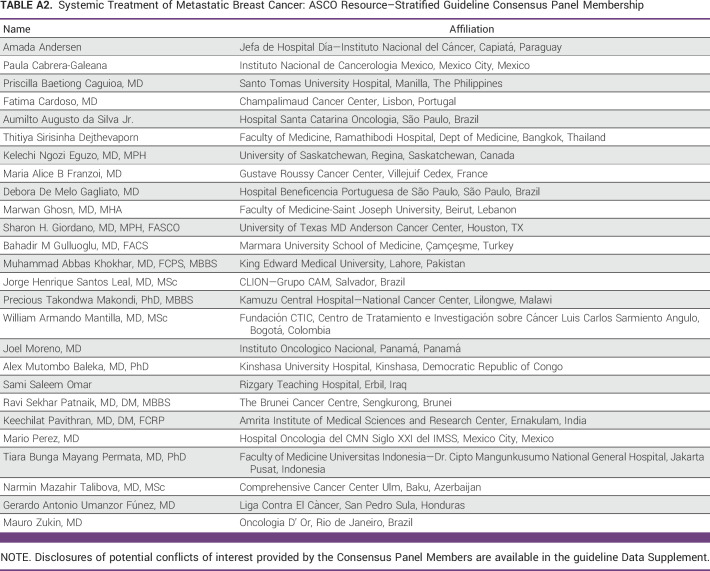
Systemic Treatment of Metastatic Breast Cancer: ASCO Resource–Stratified Guideline Consensus Panel Membership

**TABLE A3:**
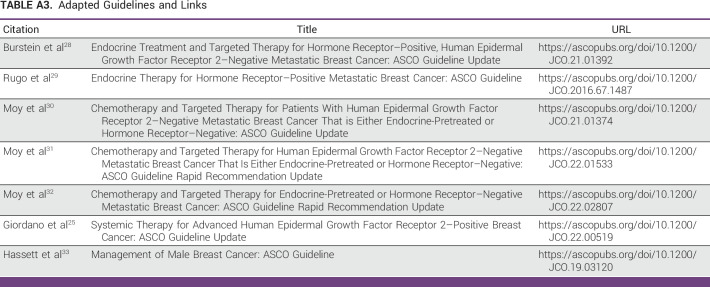
Adapted Guidelines and Links

**TABLE A4:**
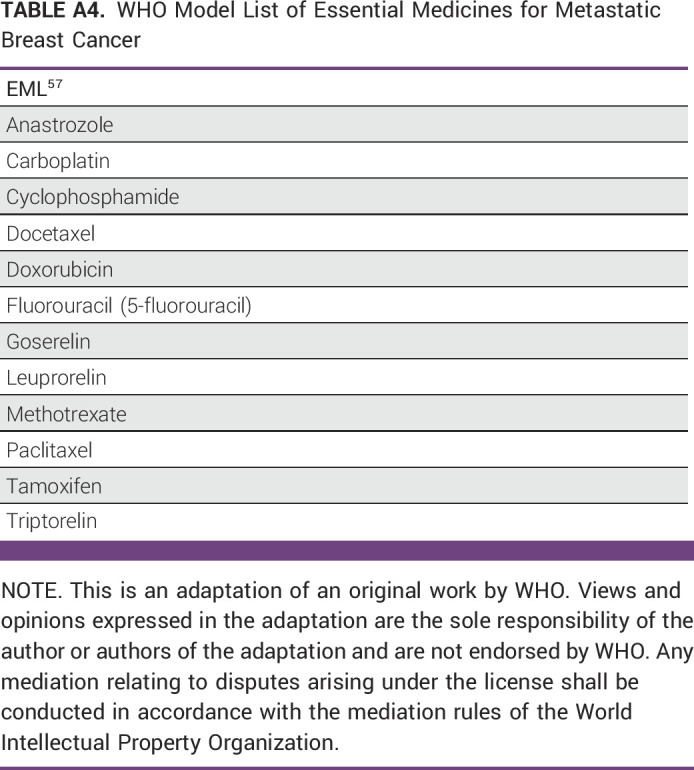
WHO Model List of Essential Medicines for Metastatic Breast Cancer

## EDITOR'S NOTE

This ASCO Guideline provides recommendations, with comprehensive review and analyses of the relevant literature for each recommendation. Additional information, including a supplement with additional evidence tables, slide sets, clinical tools and resources, and links to patient information at www.cancer.net, is available at www.asco.org/resource-stratified-guidelines.
